# Beyond G-Quadruplexes—The Effect of Junction with Additional Structural Motifs on Aptamers Properties

**DOI:** 10.3390/ijms22189948

**Published:** 2021-09-14

**Authors:** Weronika Kotkowiak, Anna Pasternak

**Affiliations:** Department of Nucleic Acids Bioengineering, Institute of Bioorganic Chemistry, Polish Academy of Sciences, Noskowskiego 12/14, 61-704 Poznan, Poland

**Keywords:** G-quadruplex, G-quadruplex junction, duplex, hairpin, triplex, aptamer

## Abstract

G-quadruplexes constitute an important type of nucleic acid structure, which can be found in living cells and applied by cell machinery as pivotal regulatory elements. Importantly, robust development of SELEX technology and modern, nucleic acid-based therapeutic strategies targeted towards various molecules have also revealed a large group of potent aptamers whose structures are grounded in G-quadruplexes. In this review, we analyze further extension of tetraplexes by additional structural elements and investigate whether G-quadruplex junctions with duplex, hairpin, triplex, or second G-quadruplex motifs are favorable for aptamers stability and biological activity. Furthermore, we indicate the specific and pivotal role of the G-quadruplex domain and the additional structural elements in interactions with target molecules. Finally, we consider the potency of G-quadruplex junctions in future applications and indicate the emerging research area that is still waiting for development to obtain highly specific and effective nucleic acid-based molecular tools.

## 1. Introduction

Aptamers are single-stranded oligonucleotides with specified sequences which determine the adoption of a strictly defined spatial structure. This enables highly specific binding to target molecules and modulation of their activity [1]. The origin of the application of nucleic acids as high-affinity ligands comes from the independent works of L. Gold and J. Szostak, where systematic evolution of ligands by exponential enrichment (SELEX) has been discussed for the first time [2,3]. This main method of aptamers development relies on the selection of oligonucleotides with the greatest affinity towards a target compound from the combinatorial library as a result of repeatable cycles of binding reactions and washing off unbound molecules [4]. The discovery of aptamers has revolutionized the world of science due to their interesting properties such as high specificity, favorable dissociation constant, and relatively facile chemical synthesis [1,4,5,6].

A significant proportion of aptamers constitute G-quadruplexes presumably due to a greater negative charge density, which is twice as for double-stranded DNA, and which could influence the enhancement of interactions with proteins [7]. What is more, G-quadruplexes constitute a structurally diverse group of compounds, what results in almost unlimited possibilities of selecting aptamers towards a great repertoire of targets. All structural arrangements of G-quadruplexes possess common feature i.e., the presence of G-tetrads formed by Hoogsteen hydrogen-bonded guanines and stabilized by cations such as K+ [8] or Na+ [9]. Two or more G-tetrads stack upon each other forming a G-quadruplexes core [9,10] connected by several types of loops, which can be divided into propeller, lateral, and diagonal [11]. Depending on the arrangement of strands within the G-quadruplexes core, three different categories of folding topologies can be distinguished: parallel (all strands have the same orientation), antiparallel (two pairs of contrary oriented strands), and hybrid (three strands have the same orientation, one strand is aligned oppositely) [10,12]. Furthermore, based on the number of strands, G-quadruplexes can be further divided into intramolecular (formed by one strand) or intermolecular (multiple strands) [10]. In order to improve aptamers biological and physicochemical properties, a great number of modifications have been introduced. These alterations concerned the substitution of nucleotides with modified residues [13,14,15,16,17,18,19,20,21,22,23], changes to the phosphodiester bond [24,25,26,27], or structural variation [28,29,30,31,32,33].

Herein, structural engineering of G-rich aptamers by the junction with various structural motifs will be discussed. This interesting approach could provide improvements in aptamers affinity towards target compounds, multivalency of binding [34], or changes in the pharmacodynamic and pharmacokinetic properties [35]. The literature data collected in this review constitute a compendium of knowledge that might be potentially useful for the development of novel molecular tools or improvements of existing aptamers utility for therapeutic applications.

## 2. Quadruplex–Duplex Junction

**Figure 1:**
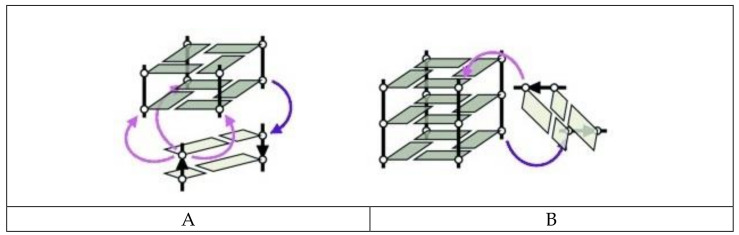
The coaxial (**A**) and orthogonal (**B**) types of quadruplex–duplex junctions with a fixed (purple) or variable (pink) point of conjugation, according to [36]. Reproduced by permission from John Wiley and Sons, © 2013 Wiley-VCH Verlag GmbH & Co. KGaA, Weinheim.

The mutual connection of G-quadruplex and the double helix is one of the most commonly described types of tetraplex junction. G-quadruplexes are characterized by various topologies depending on the relative orientation of strands within the core and the types of spanning loops. Importantly, the types of groves, i.e., wide, medium, or narrow, within the G-quadruplex core, depend on its folding topology [36]. In contrast, in a duplex structure, the minor and major grooves are determined by the alignment of two strands. Thus, the construction of a stable quadruplex–duplex junction requires certain structural compatibility because of the different dimensions of these two motifs and is possible only if the mutual connection is permissible from a thermodynamic and geometrical point of view. In theory, a number of various DNA quadruplex–duplex orientations can be distinguished (Figure 1).

The junction of antiparallel DNA G-quadruplex with the duplex domain aligned across the wide groove results in a gradual transition between the two structural elements with important stabilizing input of coaxial stacking of heterocyclic residues over the duplex and G-quadruplex motifs (Figure 1A). Interestingly, the presence of additional strand breaks within G-tracts facilitates the arrangement of duplex wide groove across the medium groove edge of G-quadruplex, thus extending the repertoire of the duplex-mediating G-rich strands orientation beyond antiparallel. In contrast, the junction of diagonal strands forming G-tetrad by helix requires an additional GA mismatch between these structural motifs. Importantly, the direct extension of a duplex across the narrow groove of G-quadruplex is energetically unfavorable due to the large, 6Å difference in dimensions of both motifs [36]. In the case of the orthogonal orientation of both structural motifs, the extrusion of base pair which joins G-quadruplex and duplex domains is observed (Figure 1B). Such disruption provides the most optimal base stacking within both motifs.

The strategic positioning of both structural elements within quadruplex–duplex junctions was adopted to improve the properties of many G-quadruplex-based aptamers with a particular emphasis on anticoagulants.

### 2.1. Thrombin-Inhibiting DNA Aptamers

The most well-known G-quadruplex-based aptamer is thrombin binding aptamer (TBA, also known as HD1). It was discovered in 1992 by L. Bock as a consequence of the canonical SELEX method [37]. TBA is a DNA 15-mer, which forms an intramolecular, antiparallel G-quadruplex with a chair-like structure. The aptamer selectively binds thrombin at exosite I (also named fibrinogen recognition exosite or anion-binding exosite), thus inhibiting fibrin clot formation. The structure of TBA is already described in detail [38,39] and is stabilized by potassium ions to provide maximal anticoagulant activity [40]. By the last years, there have been many attempts to modify the aptamer, including the substitution of natural nucleoside moieties by their analogs [41,42,43,44,45], modification of the backbone [24,46], sugar moieties [20,47,48,49,50], and cyclization [51]. Importantly, the extension of TBA leading to a quadruplex–duplex junction is one of the most common structural modifications.

**Figure 2:**
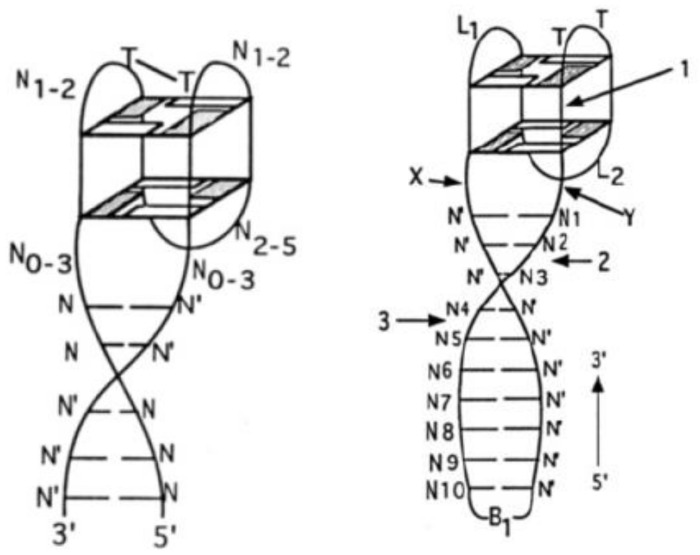
The presumed consensus structure of non-bridged (**left**) and bridged (**right**) quadruplex–duplex junctions selected by Macaya et al. [52], N—variable base position, N′—base complementary to N, B_1_—triethylene glycol/disulfide bond, grey rectangles—guanosines in *syn* conformation. Reproduced with permission from ACS. Copyright © 1995, American Chemical Society.

One of the pioneering works in the field of quadruplex–duplex aptamers was described in 1995 by Macaya et al. [52]. The authors performed a selection of DNA oligonucleotides targeted towards thrombin to investigate how the results from their SELEX would differ from the original Bock’s selection. Although they confirmed that TBA can be selected from their experiment, the vast majority of identified molecules contained additional 4–7 base pairs spanning both ends of the G-quadruplex structure and showing higher affinity to target protein than the G-quadruplex motif alone (Figure 2; Table 1, ONM). 

The topology of the G-quadruplex motif in the junctions was the same as for the isolated G-quadruplex molecule. Interestingly, the additional duplex domain was formed by 5′ATGT and 5′GTAG more often than by other sequences. Nevertheless, it was difficult to unambiguously indicate the optimal duplex motif sequence, which could be responsible for improved affinity to thrombin. NMR studies indicated that the duplex domain contributes to the overall stability of the G-quadruplex motif. Moreover, the presence of the helix might be partly responsible for the higher affinity of the aptamers to thrombin. However, no strict correlation between stability and affinity was found. The newly selected quadruplex–duplex aptamers competed with their G-quadruplex counterparts for binding with thrombin, indicating that the anion-binding exosite is the domain that interacts with the new aptamers. Importantly, the studies showed that increased affinity to thrombin of quadruplex–duplex junctions is not the result of non-specific binding but rather is a consequence of additional or improved contacts with thrombin. However, the binding affinity was not strictly correlated with anticoagulant properties of selected quadruplex–duplex aptamers and some of the molecules appeared to be poor inhibitors of fibrin clot formation regardless of high binding affinity. Nevertheless, spanning both ends of the aptamers with triethylene glycol or a disulfide bond resulted in significant improvements of their enzymatic resistance and anticoagulant properties.

**Figure 3:**
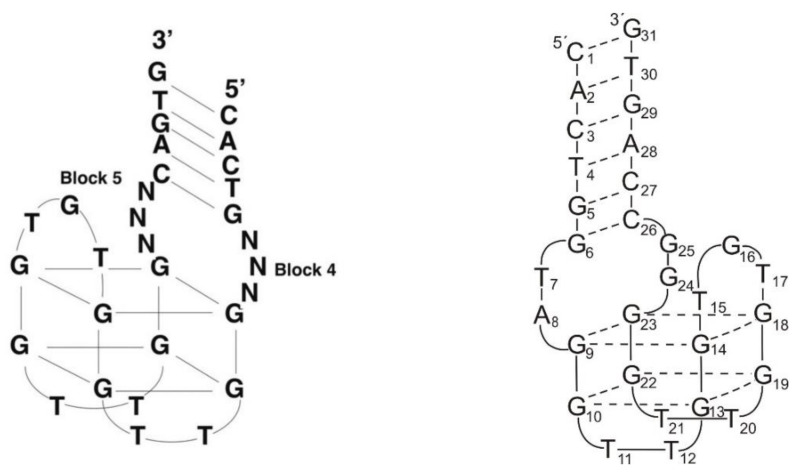
The proposed structure of quadruplex–duplex conjugates selected by in silico screening [53] (**left**) and 31TBA (**right**). The N corresponds to A, C, G, or T. Reproduced by permission from the Nucleic Acids Research and Oxford University Press.

An alternative approach to the SELEX method, using the evolution-mimicking algorithm (EMA), was proposed by the Karube group [53]. The initial in silico screening of DNA oligonucleotides as a post-SELEX optimization procedure provided 50 oligonucleotide sequences, which potentially form a quadruplex–duplex junction, i.e., 31-mer DNAs with a TBA core and 8 nt-long flanking sequences at both ends with 5–6 base-paired helix region (Figure 3, left; Table 1, ONK). Among these, only three showed improved inhibitory activity of fibrin clotting, thus suggesting that not all helix motifs increase anticoagulant properties of G-quadruplex aptamers. Moreover, further mutation of nucleotide residues that span G-quadruplex and duplex motifs within two of the most potent variants resulted in the formation of twelve new variants. The clot inhibition assay revealed that seven oligonucleotides possess improved anticoagulant properties in comparison to TBA, whereas five were worse inhibitors of thrombin activity. This suggests a critical role of the region spanning G-quadruplex and duplex motifs in the biological activity of this type of junction. One of the most potent variants, named 31TBA, with a six base-paired duplex motif and 2 nt linkers, was further structurally characterized by Dolinnaya et al. (Figure 3, right; Table 1, 31TBA) [31]. It was shown that the G-quadruplex motif of the aptamer retains intramolecular, antiparallel folding topology, which is characteristic for 15 nt TBA. Interestingly, disruption of the duplex motif by the presence of non-complementary 8 nt fragments abolishes folding of the G-quadruplex motif, whereas a lack of G-tetrads disturbs the formation of the helix domain.

**Figure 4:**
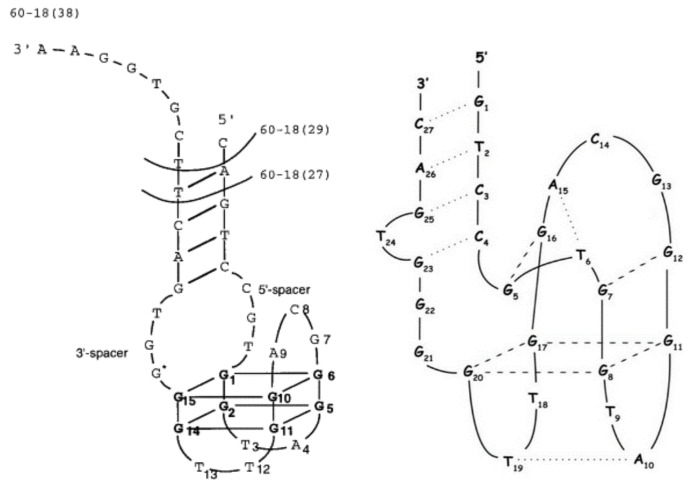
The structure of 60-18(29) (HD22(29)) and 60-18(27)) (HD22(27)) aptamer developed by Tasset et al. [54] (**left**) and HD22(27) structurally characterized by Krauss et al. [71] (**right**). Left: bold letters correspond to G-quadruplex core, G * indicates residue, which is important for efficient interactions with thrombin. Right: dotted lines—H-bonding, dashed lines—G-G Hoogsteen interactions. Reproduced by permission from Elsevier, © 1997 Academic Press Limited and IUCr Journals, © 2013 International Union of Crystallography Printed in Singapore.

Similar constructs were described by Tasset et al. (Figure 4; Table 1; HD22-29) [54]. The authors characterized a quadruplex–duplex junction with a G-quadruplex motif similar to TBA and changes in the sequence of two loops, i.e., with a T to A substitution at one of the external TBA loops (T4 position) and GCA of the central loop. Interestingly, this junction showed a 20- to 50-fold higher affinity to thrombin in reference to regular TBA, however, the clotting time was shorter than for TBA. The T to A substitution excluded T4–T13 interactions, thus leading to destabilization of the G-quadruplex structure. However, the unfavorable effect was partially compensated by the presence of an additional duplex motif. The helix domain was formed by four base pairs and attached to the G-quadruplex part via 3 nt-long linkers. The authors found no correlation between the specific sequence of the duplex domain and the inhibitory activity of the junction. The only exception was the G-C base pair at the base of the helix, which was recognized as a favorable element for substantial affinity to thrombin. On the contrary, the presence of 3 nt-long single-stranded spacers with G at position 16 seems to be preferred for high inhibitory activity. Interestingly, according to the analysis of the junction complex with thrombin, the aptamer binds with heparin-binding exosite (exosite II), which is another electropositive active site. Importantly, detailed structural studies performed by Krauss et al. of the complex between thrombin and 27-mer aptamer (60-18(27)) also named HD22(27)), which was initially identified by Tasset et al., indicates an untypical, sharply kinked architecture of the quadruplex–duplex junction (Figure 4) [71]. The spatial model of HD22(27), based on its crystallographic structure, assumes a pseudo-G-quadruplex motif linked directly to the duplex motif indicating G5 as a pivot residue, which mediates the shift from duplex to the G-quadruplex domain. Noteworthy, the helical axes of both motifs are oriented almost perpendicular to each other. Such an arrangement facilitates optimal contact of both domains of the aptamer with exosite II of thrombin. Furthermore, interactions of HD22(27) with the target molecule increase the thermal stability of the aptamer.

**Figure 5:**
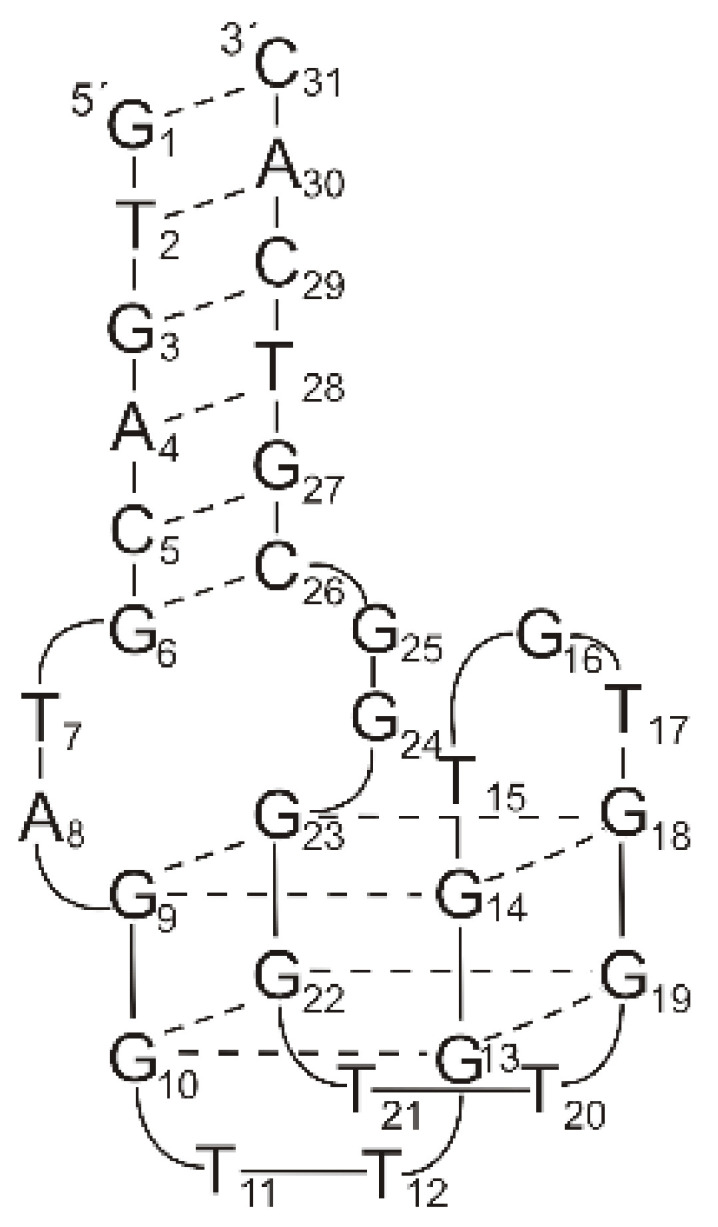
The structure of the RE31 quadruplex–duplex junction.

In 2011 Mazurov et al. characterized a new antithrombin aptamer named RE31 [30]. The molecule consists of a 15 nt-long TBA G-quadruplex motif linked by two 2 nt spacers with a 6 bp duplex domain (Figure 5; Table 1, RE31). It shows improved affinity towards thrombin and significantly prolonged clotting time in comparison to TBA. Moreover, RE31 was found to have a species-specific effect on the target protein by inhibiting the activity of human thrombin more than rat and rabbit thrombin. Interestingly, the presence of the duplex motif destabilizes the structure of the G-quadruplex domain [72]. However, the studies performed for truncated RE31 variants suggest that the stability of the G-quadruplex motif is directly proportional to the number of base pairs within the helix part of the quadruplex–duplex junction. The authors also suggested a direct correlation between aptamer stability and its affinity to thrombin. However, the article published in 2015 does not confirm this finding [55]. Nevertheless, RE31 remains the most potent anticoagulant among all truncated aptamer variants presumably due to additional contacts provided between the quadruplex–duplex junction and target protein. The above studies suggest that G-quadruplex is a functional domain in the thrombin–aptamer interactions, whereas the duplex motif facilitates favorable conformational adjustment of the molecule. Moreover, it seems that the length of the duplex motif is not the only factor that influences aptamers affinity to thrombin, since variants with AT-closing base pairs show lower affinity to target molecule than ones with GC base pairs at the end of the helix [55]. According to X-ray studies, both structural motifs of RE31 are coaxially stacked on top of each other due to the gradual transition between the helix and G-quadruplex domains [32]. Importantly, the TGT loop mediates in this junction by the formation of hydrogen bonds with linker residues (i.e., GG and TA) resulting in the formation of three base pairs. The perfectly stacked domains in the overall structure of RE31 prevent interactions of both motifs at once with the same protein molecule. Indeed, the contact with thrombin exosite I was observed only for the G-quadruplex part of the aptamer. However, improved binding affinity is probably due to interactions of RE31 with three various regions of thrombin, and presumably also due to the involvement of the TGT loop in the additional interactions, which might increase enzymatic stability of the whole aptamer. 

The attempts of Kotkowiak et al. to characterize chemically modified variants of RE31 indicate no strict correlation between aptamers thermal stability and inhibitory activity of fibrin clotting [23]. The aptamer was modified with locked nucleic acid (LNA), unlocked nucleic acid (UNA), and β-L-RNA nucleoside residues resulting in a series of 13 novel molecules. Although the authors noticed that the most thermally stable variants containing, simultaneously, LNAs in the duplex motif and single UNA within the G-quadruplex domain showed a 2-fold improved inhibitory activity of fibrin clotting, the aptamer modified exclusively with LNAs possessed anticoagulant potential comparable to unmodified RE31 regardless of an almost 11 °C higher melting temperature.

**Figure 6:**
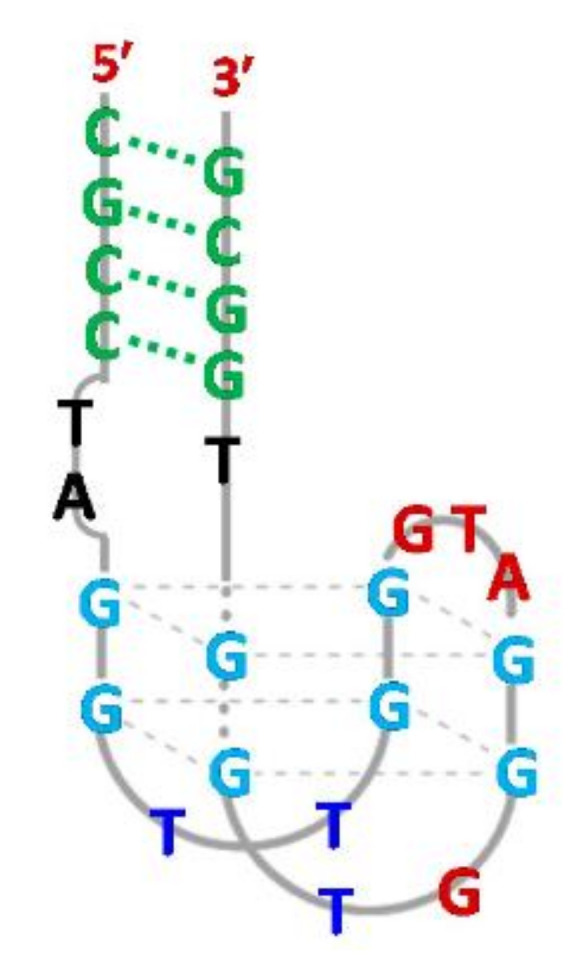
The structure of NU172 [75]. Reproduced under the Creative Commons Attribution License.

Currently, the only quadruplex–duplex junction which has entered Phase II clinical trials is NU172, also known as ARC2172 (Figure 6; Table 1, NU172). This aptamer is a DNA 26 mer with significantly improved affinity to thrombin and 1.5 times higher anticoagulant potency in reference to TBA [73]. Although the end of the Phase II trial was planned in 2013, the current status of the study is unknown [74]. NU172 interacts with exosite I of thrombin similar to TBA. According to crystallographic studies, the aptamer folds into a quadruplex/duplex junction with continuous stacking of residues within both structural motifs. The sharp transition between the two domains is assured due to the presence of reverse Hoogsteen interactions between the GTA loop within G-quadruplex and TAT residues from linkers connecting both domains [56]. Moreover, the inter-domain region allows interactions between the GTA loop and target protein. Thus, efficient contact of NU172 with thrombin requires mutual interactions of a few structural elements, i.e., the presence of G-quadruplex and duplex domains spanned together in a rod-like architecture via a TAT triad, two guanine residues in the G-quadruplex loops, which additionally locks the G-quadruplex structure, and finally, the G-G base pair formed between loops. An interesting attempt to improve NU172 by chemical modifications was made by the Montesarchio group [75]. The cyclic derivatives of NU172 showed unchanged folding topology in comparison to the unmodified aptamer, higher thermal, and enzymatic stability, but all of the variants appeared as worse anticoagulant agents than NU172. The above results indicate no strict correlation between the aptamer thermal stability and inhibitory activity. Presumably, the presence of a flexible linker spanning both ends of NU172 might affect the architecture of the quadruplex–duplex junction, thus also the G-quadruplex loop spatial arrangement, and in such a way affects thrombin recognition.

Interestingly, the comparative structural studies of four quadruplex–duplex junctions, i.e., RE31, HD22 (27 mer and 29 mer), and NU172 revealed that such constructs change conformation depending on the type of monovalent cations with the exception of RE31 [57]. Furthermore, NU172 and RE31 unfold in potassium buffer in one step what is probably due to the existence of two different structural types, i.e., sharply kinked and rod-like architectures, whereas two remaining junctions unfold according to a two-step mechanism. Noteworthy, in the case of NU172 and RE31, the duplex motif might counterbalance the energetically unfavorable effect of sodium ions.

### 2.2. Another Quadruplex–Duplex Junctions

Despite a large number of articles concerning anticoagulant quadruplex–duplex junctions, some G-quadruplexes structural functionalization with a helix domain has been described to obtain highly selective aptamers towards targets other than thrombin. 

Recently, a characteristics of novel quadruplex–duplex junction based on AS1411 aptamer was published (Table 1, AS1411-N6) [28]. AS1411 is the most clinically advanced antiproliferative and pro-apoptotic aptamer, which forms a highly polymorphic 26 nt-long DNA G-quadruplex structure with the majority of topologies in parallel strand orientation [76,77]. It shows promising biological activity by specific interaction with nucleolin, which is expressed on the surface of a great number of various cancer cell types. Direct junction of AS1411 with six base-paired helix region results in a decrease in the structural polymorphism of the G-quadruplex motif presumably by locking the dynamic AS1411 structure [28]. On the other hand, the junction is less thermally stable than parental G-quadruplex and shows decreased binding affinity towards nucleolin.

Interestingly, one TBA junction (31TBA, Figure 3) was used in the development of novel aptasensors with a dye-forming cavity between two domains [78]. The authors characterized properties of variants with a helix domain of varying lengths, i.e., truncated and elongated, and found the aptasensor, which lights up 7–20 times in comparison to parental G-quadruplex. The analysis of interactions of GFP chromophore analogues with 31TBA and its variants revealed that increased fluorescence intensity can be achieved in the case of shorter duplex motifs joined to the G-quadruplex domain, whereas the presence of an elongated helix domain leads to a decrease in the fluorescence signal. The above effects might be explained by modulation of the junction structure rigidity, which increases with helix domain length, hindering the optimal positioning of dye, or by nonspecific interactions of dye with the duplex motif with no fluorescence signaling. In contrast, the topology of G-quadruplex does not influence the selective enhancement of fluorescence intensity.

**Figure 7:**
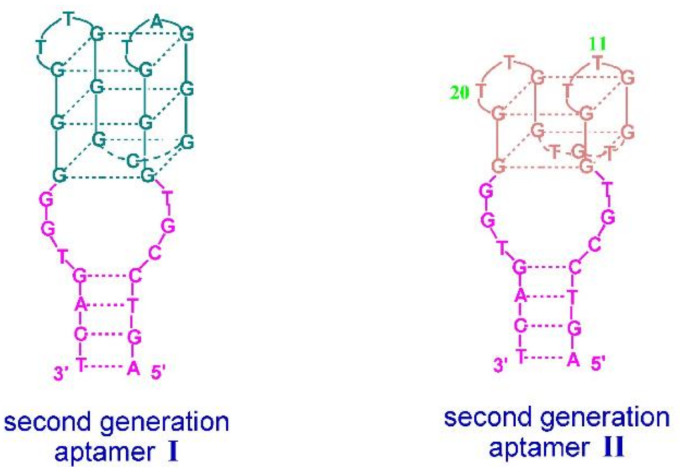
Schematic structure of quadruplex–duplex junctions with peroxidase-like activity [58]. Reproduced under the Creative Commons Attribution License.

The presence of a duplex domain appeared favorable also for the improvement of the properties of G-quadruplex aptamers targeted towards hemin, resulting in the development of a new type of DNAzymes with peroxidase-like activity. The grafting strategy proposed by Li et al. provided quadruplex–duplex junctions with increased binding affinity in comparison to the initial G-quadruplex aptamer [58]. The first molecule is formed by antiparallel DNA G-quadruplex joined via 3 nt linkers with four base-paired helix domains (Figure 7; Table 1, Aptamer I). The affinity of the construct to hemin is moderately improved in comparison to the parental G-quadruplex. However, evaluation of DNAzyme activity of aptamer I by analysis of the oxidation product revealed a 35% improvement which is attributed to the presence of the duplex motif as well as a specific sequence of 3 nt linkers. The second junction studied by Li et al. was formed based on TBA G-quadruplex which was elongated by the same duplex domain as in the case of aptamer I (Figure 7; Table 1, Aptamer II). Interestingly, this construct showed not only four times improved binding affinity to thrombin, but also possessed the ability to bind hemin. All the above findings confirm that an additional helix domain with an appropriate linker sequence contributes to the improvement of the affinity of an aptamer to a target molecule and to the formation of peroxidase-like DNAzyme.

**Figure 8:**
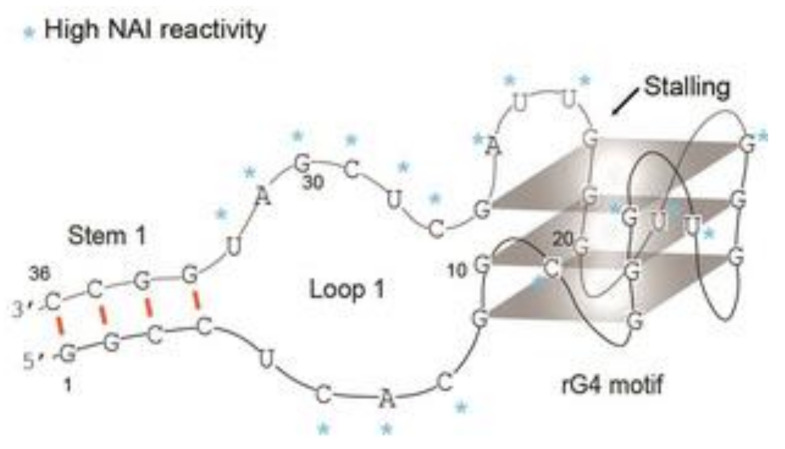
The proposed structure of Ap3-7 [79]. Reproduced with permission from John Wiley and Sons, © 2020 Wiley-VCH Verlag GmbH & Co. KGaA, Weinheim.

An untypical example of a fully modified quadruplex–duplex junction is Ap3-7, which was described in 2020 as the first Spiegelmer (aptamer composed of β-L-RNA residues), targeted towards biologically relevant, non-coding telomeric repeat-containing RNA (*TERRA*) G-quadruplex fragment [79]. The aptamer was developed via in vitro selection and consists of a G-quadruplex motif linked by single-stranded fragments of different lengths (4 and 6 nt) with a four base-paired duplex domain (Figure 8). The extended studies showed that interactions between the junction and target G-quadruplex are strong and enantiomer-specific, i.e., that an L-RNA type aptamer binds only a D-RNA type target. Moreover, the aptamer efficiently interfered with RHAU53 peptide (fragment of RNA helicase associated with AU-rich element) binding to *TERRA* G-quadruplex, showing an interesting alternative in designing novel molecular tools of therapeutic importance selectively targeted towards naturally occurring G-quadruplex targets.

**Figure 9:**
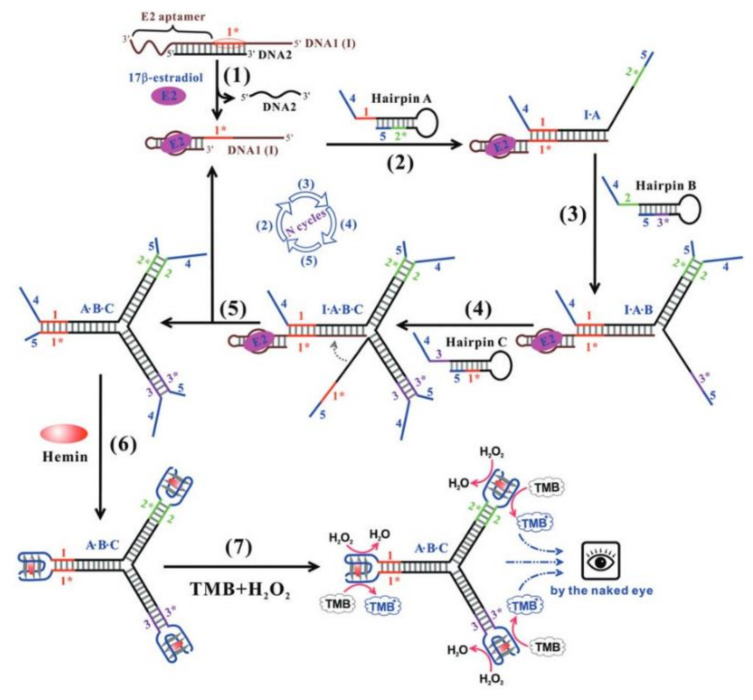
Scheme of diagnostic platform for detecting 17β-estradiol [80]. Reproduced with permission from Royal Society of Chemistry (Great Britain), © The Royal Society of Chemistry 2015.

The application of a quadruplex–duplex junction as a diagnostic platform for detecting 17β-estradiol is also worth discussing. The system is composed of three probes (Figure 9, Hairpin A–C), which form target-induced three-way quadruplex–duplex junctions with non-covalent linkages [80]. In this arrangement, each probe simultaneously contains half of the G-quadruplex and duplex sequences from two arms of the structure, and only after complete hybridization is the whole platform functional. The detection rests upon hemin-induced peroxidation activity of G-quadruplex fragments, which are DNAzymes able to catalyze the H_2_O_2_-mediated oxidation of 3,3′,5,5′-tetramethylbenzidine (TMB) (Figure 9, Steps 6 and 7). Importantly, the additional elements of the system are two complementary oligonucleotides. The first one is able to bind 17β-estradiol and one of the probes (Figure 9, DNA 1). This binding is the initial step in the three-way quadruplex–duplex junction formation. The second strand forms a duplex with the first oligonucleotide in the absence of the ligand and masks its activity (Figure 9, DNA 2). The above is a perfect example of the practical usage of a quadruplex–duplex junction for the detection of organic compounds and application as an aptasensor platform without the need of labeling and immobilization of probes. 

## 3. Quadruplex–Quadruplex Junction

An interesting example of chemical alteration of G-quadruplex structures is the development of conjugates composed of two G-quadruplex particles, termed bivalent aptamers. The components can have different targets, but more frequently it is a combination of the compounds targeted towards different binding sites of the same molecule. This approach enhances the bivalency of conjugates what ensures higher binding affinity in comparison to parental compounds, which is a result of multiple binding events, a higher rebinding rate, and, above all, better target selectivity [34]. 

One of the first attempts of combining two G-quadruplexes was performed for thrombin binding aptamers [59]. In this research, a group of conjugates formed by 15 nt-long TBA covalently connected via linkers containing 5 to 10 units of spacer18 with a 29 nt-long compound, named 60-18(29) (HD22(29)) [60], was synthesized and characterized (Table 1, THR5-7). As discussed in a previous paragraph, the 60-18(29) adopts a quadruplex–duplex structure and in contrast to TBA, binds to thrombin heparin-binding exosite [54]. The different binding sites of two G-quadruplex domains within conjugates resulted in the cooperativity of their action, which was reflected in the increased affinity towards thrombin [59]. The best variant, THR7, which is composed of TBA and 60-18(29) connected by a 10-unit-long linker, was the most efficient competitor to wild-type TBA and entirely inhibited its binding to thrombin at a 1:1 ratio. Based on the above, it was estimated that the Kd value of THR7 is below 97 pM, and its binding affinity is 65-fold higher than TBA. The detailed analysis of the data also revealed that the appliance of a longer linker (10 vs. 5 units) and specific order of components (TBA aptamer localized at the 5′-end of the conjugate) resulted in a more favorable affinity towards thrombin. 

**Figure 10:**
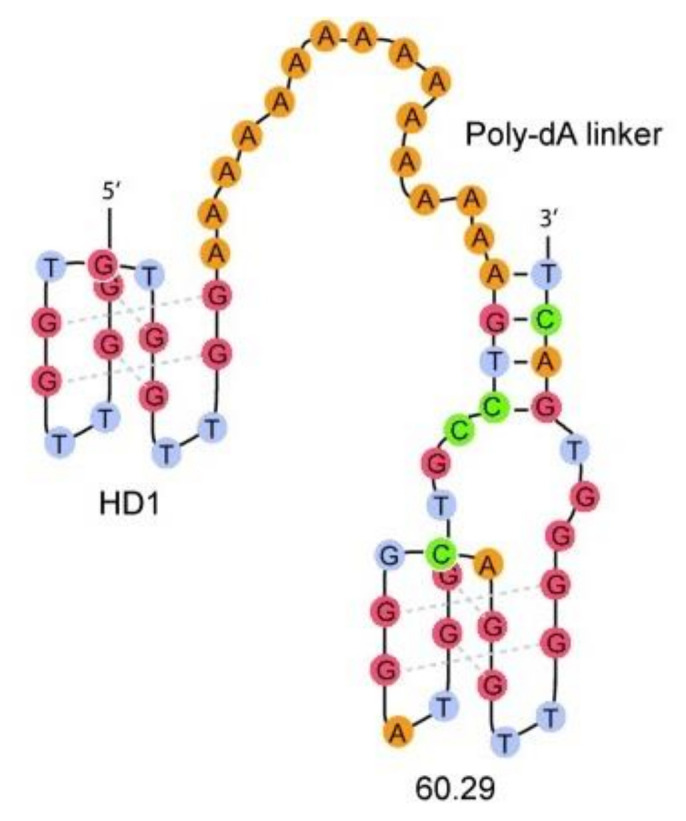
The structure of bivalent aptamer composed of HD1 (localized at the 5′ end) connected with 60.29 (HD22(29)) via a poly-dA linker [81]. Reproduced with permission from John Wiley and Sons, © 2007 Wiley-VCH Verlag GmbH & Co. KGaA, Weinheim.

The above system was also used by the Mayer group, however, the spacer18-based linker was replaced by a poly-dA linker (Figure 10) [81]. The main goal of this approach was to increase the affinity of the conjugate via additional electrostatic interactions between the poly-dA linker and positively charged amino acid residues localized in close proximity to exosite I and II. Similar to previous research, two lengths of a linker, 5 and 15 nt long were used (Table 1, HD1-5dA-60.29 and HD1-15dA-60.29). Based on the data analysis, it can be concluded that the application of a longer linker (HD1-15dA-60.29) is more favorable for increasing the biological properties of the conjugate. A significant improvement in the affinity of a bivalent aptamer toward the thrombin molecule and prolongation of clotting time with a simultaneous 30-fold decrease in dose was observed. What is more, the conjugate exhibited an action profile similar to a commonly used anticoagulant agent (bivalirudin), with the advantageous possibility of inhibition its action via the application of complementary oligonucleotide as an antidote [60]. 

**Figure 11:**
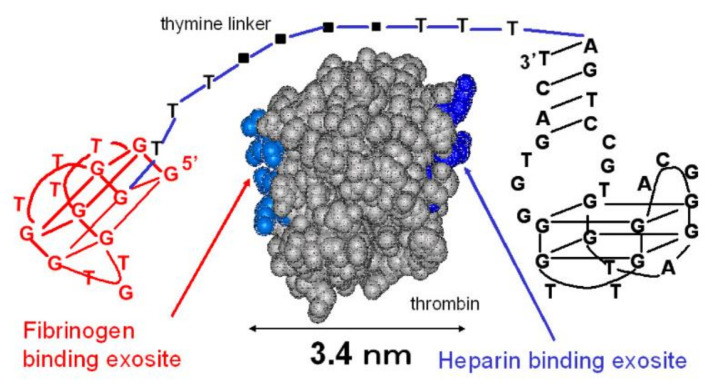
The structure of bivalent aptamer composed of HD1 (located at the 5′ end) connected with HD22(29) via a poly-dT linker [61]. Reproduced under the Creative Commons Attribution License.

Almost simultaneously, Ikebukuro and co-workers developed a compound also composed of HD1 and HD22(29) aptamers but linked by poly-dT linkers with lengths ranging from 5 nt to 20 nt [61] (Figure 11; Table 1, Linker 0–20). The observed biological parameters of conjugates were not entirely consistent with the previous studies. Although the bivalent aptamer connected by the longest 20 nt-long linker (Linker 20) turned out to be the most effective thrombin activity inhibitor out of the whole group of compounds, its Kd value was one of the highest. On the contrary, the most favorable Kd value was observed for the bivalent aptamer connected by only five thymidine residues (Linker 5). The above results can be explained by the fact that all Kd values were in the subnanomolar range. Therefore, the differences in the affinity were minor and the disturbance of fibrinogen binding to the thrombin molecule as an effect of spatial hindrance caused by 20 nt-long linkers in comparison to 5 nt long spacers seems to be a more important factor, which influenced bivalent aptamer activity. 

The potency of the junction between HD1 and HD22(27) in thrombin inhibition was also noticed by Kim et al. [34]. The group analyzed the system in which the aptamers were connected by 4, 6, 8, or 10 spacer phosphoramidites (named S(4-10)T; Table 1, Bi-4S to Bi-10S). The authors concluded that the anticoagulant activity of conjugates increases proportionally to the length of the linking fragment to reach the highest value for an 8 nt-long spacer (Bi-8S). After that, a drop in the inhibitory potential of bivalent aptamer (Bi-10S) was observed, but the effect was still a few orders of magnitude higher than for the wild-type TBA. Importantly, the S4T spacer, which was supposed to fully cover the distance between exosites I and II, caused a reduction in the anticoagulant activity of conjugate (Bi-4S) in comparison to the parental compound. The observed result can be justified by the need of suitable linker length to permit the appropriate orientation of both G-quadruplex domains in their binding sites with minimization of linker–thrombin contact. The shortest linker was not long enough to let both G-quadruplex motifs adhere to thrombin in the most optimal position, simultaneously reducing the effect of the linker presence. 

Further continuation of the research trend concerning complexation of HD1 with HD22(29) was performed by the Veedu group, who developed conjugates composed of the above aptamers linked by triethylene glycol (Table 1, RNV220) or four 2′-deoxythymidines (Table 1, RNV220T) and with an inverted polarity of 2′-deoxythymidine at the 3′-end [62]. Both of them were characterized by improved anticoagulant properties in comparison to wild-type TBA, but RNV220 possessed a superior ability to inhibit thrombin activity. The presence of inverted pyrimidine nucleoside residue at the 3′-end of the oligonucleotide has a protective effect, what found reflection in the extended biostability of analyzed bivalent aptamers. What is more, the anticoagulant effect of RNV220 and RNV220T could be reversed entirely due to the addition of oligonucleotide with a sequence complementary to conjugates. 

**Figure 12:**
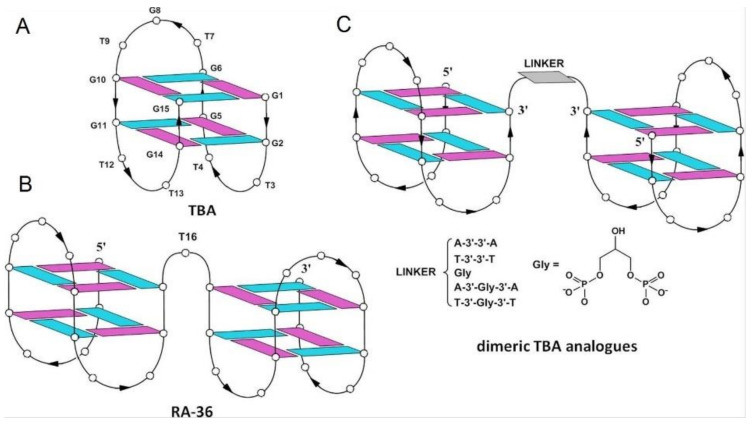
The structure of TBA (**A**), RA-36 (**B**) and bivalent aptamer composed of two HD1 domains (connected via different linkers) (**C**) [64]. Reproduced under the Creative Commons Attribution License.

Another interesting concept in the exploration of the potency of G-quadruplex complexation was the construction of a homodimer, termed RA36, composed of two HD1 motifs covalently linked by one thymidine residue (Figure 12B) [63]. One HD1 motif is responsible for ensuring the inhibitory activity of the conjugate, whereas the other modulates the action of the first one. The bivalent aptamer was characterized by comparable to the RE31 anticoagulant profile of action in in vitro blood tests [63]. What is more, it has been proven that RA36 could also exert an antiproliferative effect towards certain cancer cell lines, e.g., HeLa, RL-67, and U87 [82]. Further modification of RA36 relied on changes of component orientation and connection of both HD1 motifs at their 3′-termini via adenosine or thymidine residues and/or a glycerol (Gly) moiety, Figure 12C (Table 1, AA to TGlyT) [64]. The thermal stability of resultant variants was similar to wild-type TBA (T_m_ for variants was in range from 33 to 35 °C, T_m_ for TBA was equal to 33 °C) and slightly better than for unmodified RA36 (T_m_ was 32 °C). The two opposite exceptions constituted variants possessing linkage composed of two adenosine residues (AA) and thymidine residues with a Gly moiety (TGlyT). The quadruplex–quadruplex junction mediated by AA was characterized by a melting temperature (37 °C) that was higher than for others, whereas the one linked by TGlyT showed the lowest thermal stability (T_m_ equal to 29 °C). Moreover, the variant which contained AA also possessed the most favorable inhibitory properties and the highest biostability. The improvement in thrombin inhibition, in comparison to wild-type TBA, was also observed for junctions with TT and AGlyA linkers. Based on CD spectra analysis, the authors concluded that G-quadruplex motifs within all analyzed variants preserved an antiparallel G-quadruplex structure. Importantly, it was also claimed that the type of linker had an influence on the biostability and that the variants containing a glycerol moiety were more prone to enzymatic degradation. 

**Figure 13:**
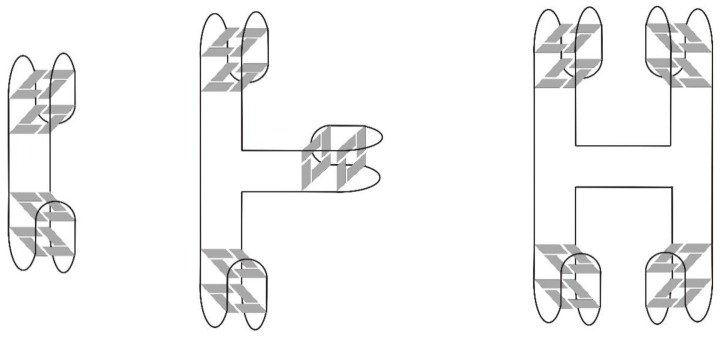
Schematic presentation of circular quadruplex–quadruplex junctions based on studies by Di Giusto and King [65].

An innovative approach was proposed by Di Giusto and King, who performed a complexation of two or more G-quadruplexes via circularization (Figure 13) [65]. The model compound constituted an inter alia GS-522 DNA aptamer (Table 1), whose homo- and hetero-dimeric, trimeric, and tetrameric constructs with HD22(29) were created. The resultant dimeric circular conjugates were characterized by a significant increase in thermal and biological stability. The heterodimers possessed almost two orders of magnitude higher anticoagulant properties than the parental GS-522 compound (Table 1, cTU1 and cTU2). The range of improvement was even larger when the circular bimodular aptamer was composed of two GS-522 molecules, (Table 1, cTT-2). As it could be predicted, further enhancement of the inhibitory activity of conjugates was observed along with the complexation of a higher number of exosite I binding domains (Table 1, cTTTT-1). 

Another interesting example of the quadruplex–quadruplex junction is the derivative of the R12 aptamer named R12-A-R12 (Table 1) [67,83]. The parental R12 is an RNA aptamer, forming a parallel G-quadruplex structure, targeted towards normal, physiological cellular prion protein (PrP^C^) and preventing its transition into an abnormal form (PrP^Sc^). The R12-based G-quadruplex junctions were composed of two R12 aptamer molecules (Table 1) connected covalently via a nucleotide bond or an additional adenosine residue, respectively. Based on NMR analysis, it was concluded that two R12 molecules form a homodimer, which is essential for tight binding of PrP^C^ but is also labile in a cellular environment. The covalent complexation of two R12 molecules resulted in the enhancement of aptamer inhibitory potential and increased the affinity of the junctions towards PrP^C^ with maintaining the R12 mode of action. In addition, the covalent linkage of two R12 molecules facilitated the preservation of the structure which is required for strong PrP^C^ binding in a cellular environment. 

**Figure 14:**
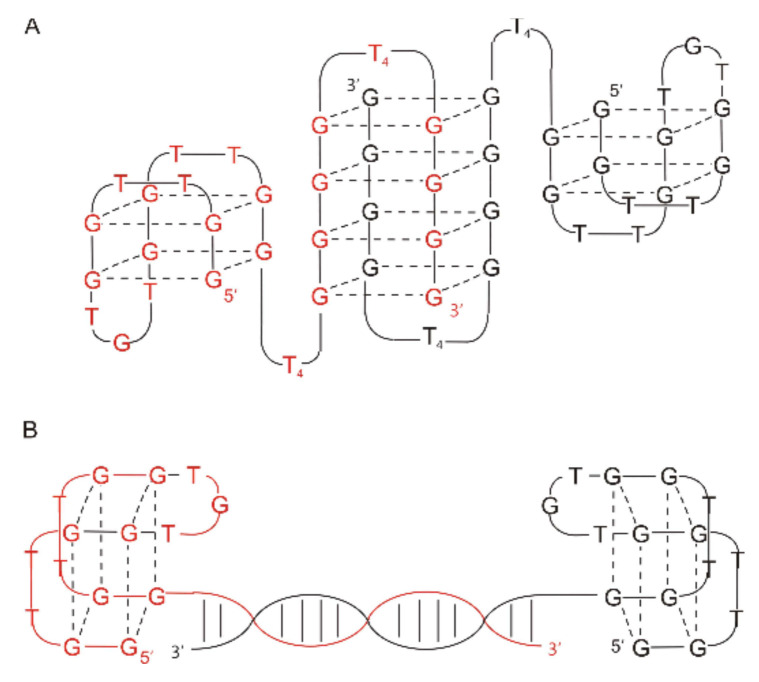
The structure of T4T4 (**A**) and HD1-ds (**B**) [35].

Apart from the covalent junction, the bivalency in aptamers constructs could also be obtained by non-covalent assembly [35,84]. In this arrangement, two HD1 particles were connected via locking of the G-quadruplex (Table 1, T4T4) or helix lock (Table 1, HD1-ds), formed between 3′-terminal fragments of each component and stabilized by Hoogsteen and Watson–Crick base pairing, respectively (Figure 14A,B). The data analysis revealed that although both conjugates showed reduced stability in thermodynamic studies in comparison to wild-type HD1 (T_M_ for HD1, HD1-ds, T4T4 was 38.8 °C, 33 °C, and 35.6 °C, respectively), they were characterized by superior affinity toward thrombin (K_D_ for HD1, HD1-ds, T4T4 was 2.6, 1.5, and 0.62 nM, respectively). Interestingly, all tested molecules displayed a similar activity level in functional activity tests (thrombin time test), which was slightly lower than for HD1. Noteworthy, the comparison of anticoagulant properties of HD1-ds with RA-36 revealed the differences in their mode of action. RA-36 was characterized by a lower inhibitory activity than HD1-ds, therefore, it could be concluded that HD1 motifs in this conjugate act as one unit and that the presence of covalent linkage causes a decrease in anticoagulant properties. On the contrary, only a small variation in thrombin inhibition in comparison to HD1 was observed for HD1-ds. Thus, it could be assumed that its components functioned as two independent domains and their activity depends only on the accessibility of exosite I. The CD analysis indicated that both conjugates exhibited features of antiparallel G-quadruplexes. Additionally, the spectrum of HD1-ds possessed also a minimum at 245 nm, which is typical for DNA duplexes.

**Figure 15:**
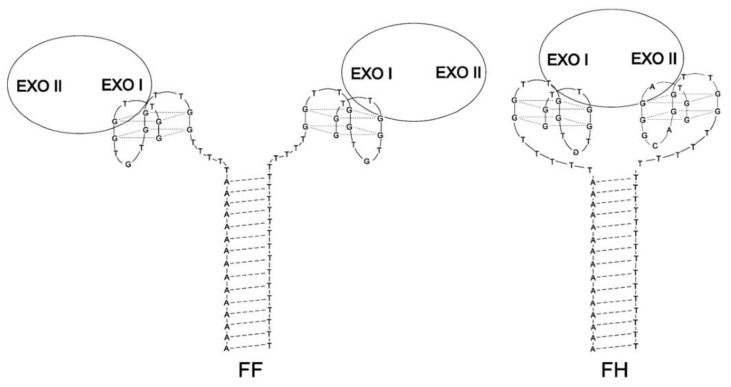
The structure of FF (**left**) and FH (**right**) dimers [66]. The FF dimer binds two thrombin molecules in their exosite I, whereas the FH dimer interacts with one thrombin molecule, but in two different binding sites: exosite I and II. Reproduced with permission from Elsevier, © 1997 Academic Press Limited and IUCr Journals, © 2013 International Union of Crystallography Printed in Singapore.

The concept of aptamers junction via hybridization was also described by Poniková et al. [66]. They have designed homo- (FF, Table 1) and heterodimers (FH, Table 1) of HD1 and HD22 connected via a 15 nt-long duplex fragment and 5 nt-long thymidine linkers (Figure 15). Based on the melting analysis, it could be assumed that the formation of dimers had a minor influence on the stability of the G-quadruplex domains (T_M_ for HD1 and HD22 with an additional single-stranded part was 40.5 °C and 44.3 °C, respectively vs. T_M_ for FF and FH was 38.1 °C and 40.1 °C, respectively), simultaneously with a slightly stabilizing effect on the duplex fragment (T_M_ for duplex alone was 46.3 °C vs. T_M_ for the duplex fragment in FF and FH was 47.7 °C and 48.5 °C, respectively). Interestingly, despite lower thermal stability, both dimeric aptamers were characterized by a significant improvement in anticoagulant properties in comparison to the parental HD1. In addition, the inhibitory activity was strongly dependent on the presence of potassium ions. It is worth noting that the above system was also used for the development of an aptasensor platform dedicated to thrombin detection, where dimeric aptamers were immobilized on a gold surface covered by neutravidin [85]. The only difference relied on the variation of the duplex and linkage fragment length (the helical part was 18 nt long, whereas linkers were 9 nt long and 21 nt long). In both examples, the improvement in the thrombin detection level in comparison to immobilized parental HD1 could be observed. 

## 4. Quadruplex–Hairpin Junction

**Figure 16:**
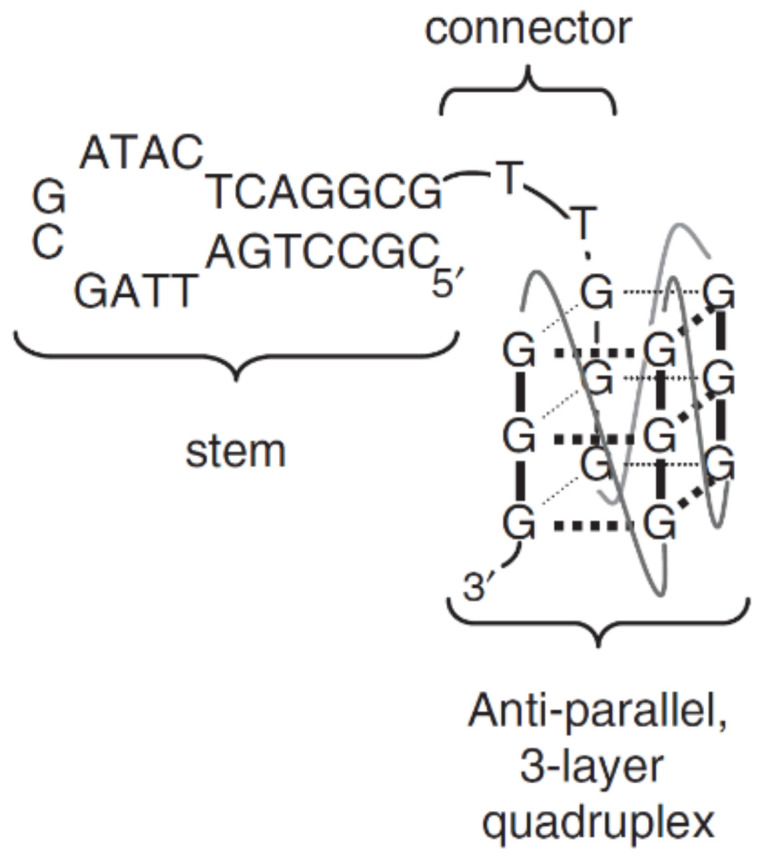
Schematic structure of quadruplex-hairpin junction [68]. Reproduced with permission from the Nucleic Acids Research and Oxford University Press.

Another interesting type of combination of G-quadruplexes with different structural motifs is their junction with a hairpin. One of the first reports on this topic concerns a group of DNA aptamers, which are inhibitors of HIV-1 reverse transcriptase (RT), called RT5, RT6, and RT47 (Table 1) [68]. All conjugates have a similar structural pattern with a stem–loop structure at the 5′-end connected by a linker (two thymidines) to the G-quadruplex motif, built up with three G-tetrads, Figure 16. It was proven that both of the above structures, connected covalently, are required to obtain the inhibitory activity of the aptamer. Moreover, it was pointed out that not the sequence of the stem fragment but rather its length could have an influence on conjugate activity. Interestingly, the types of junctions with G-tetrads also did not affect the aptamer inhibitory properties, since the presence of closed-loop and unlinked 5′- or 3′-ends was equally efficient.

**Figure 17:**
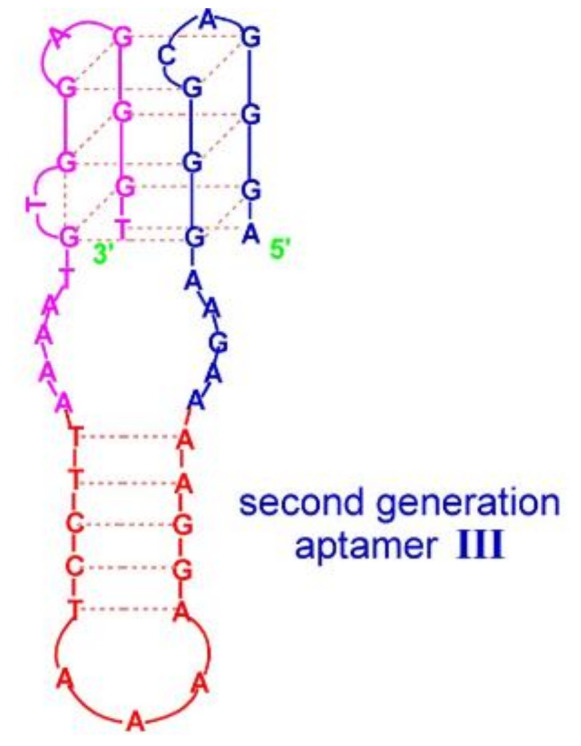
Schematic structure of quadruplex–hairpin junction with peroxidase-like activity [58]. Reproduced under the Creative Commons Attribution License.

The second example of a G-quadruplex-hairpin junction constitutes the aptamer targeted towards hemin, being a DNAzyme with peroxidase-like activity [58]. The conjugate is formed by a G-quadruplex joined by unpaired 5 nt regions with a hairpin with a five base-paired stem and a 3 nt loop (Figure 17; Table 1, Aptamer III). This quadruplex–hairpin combination also shows improved affinity in comparison to the parental G-quadruplex and higher DNAzyme activity, which seems to be due not only to the presence of the stem–loop structure but also to the type of residues joining both domains. 

**Figure 18:**
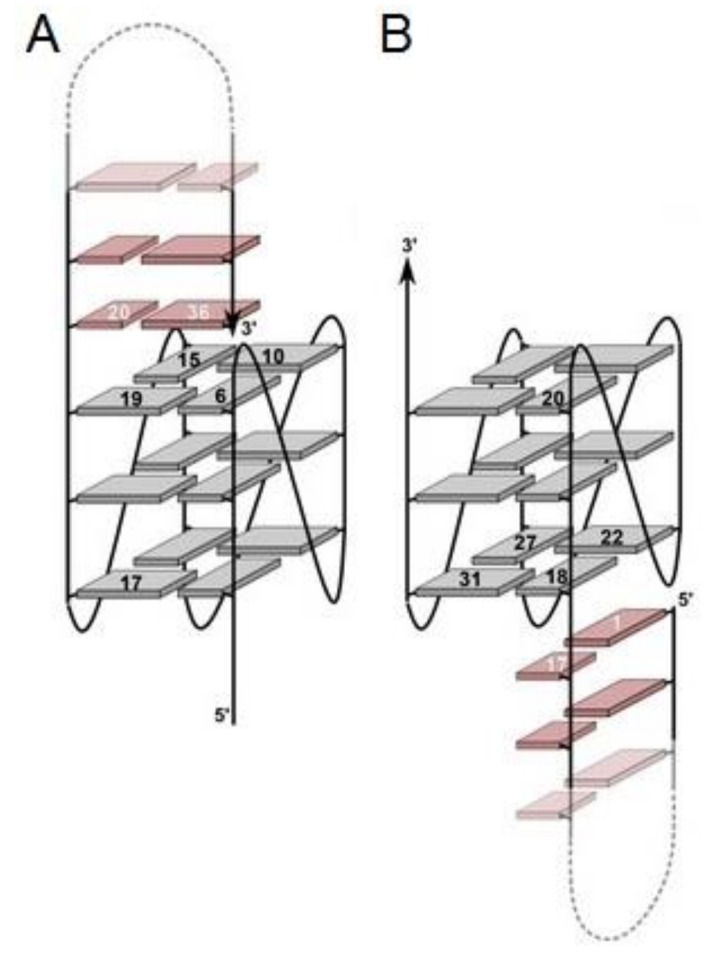
Schematic structure of Myc-dup3 (**A**) and Myc-dup5 (**B**) [69]. Reproduced under the Creative Commons Attribution License.

A great supplement for the reports referring to aptamers with quadruplex–hairpin junctions is research concerning the structural analysis of model fragments representing genome sequences, which could provide some general information about the influence of a hairpin on the G-quadruplex structure. The Weisz group created quadruplex–hairpin conjugates based on the promoter region of *c-Myc* oncogene and a hairpin fragment localized at its 3′-termini (Table 1, Myc-dup3) or 5′-termini (Table 1, Myc-dup5), Figure 18 [69]. The CD and thermodynamic data analysis showed no influence of the presence of a stem–loop structure on general G-quadruplex folding topology and stability. Interestingly, it was observed that the stem–loop structure presented at the 5′-end of G-quadruplex was more thermodynamically stable (56.1 °C, Figure 18A) than the same sequence localized at the 3′-end (45.1 °C, Figure 18B). It was also proved that a quadruplex–hairpin junction can constitute a good binding site for ligands with G-quadruplex specificity, sometimes even more efficient than the G-tetrads itself like in case the of the indoloquinoline ligand.

## 5. Quadruplex-Triplex Junction

Among a great variety of secondary structures of nucleic acids, which were combined with G-quadruplexes, triplexes also took a place. They constitute a triple-helical framework composed of a homopurine/homopyrimidine Watson–Crick duplex with a third oligonucleotide strand (TFO, triplex-forming oligonucleotide) which interacts via Hoogsteen (parallel triplex) or reverse-Hoogsteen (antiparallel triplex) hydrogen bonds [70,86]. 

**Figure 19:**
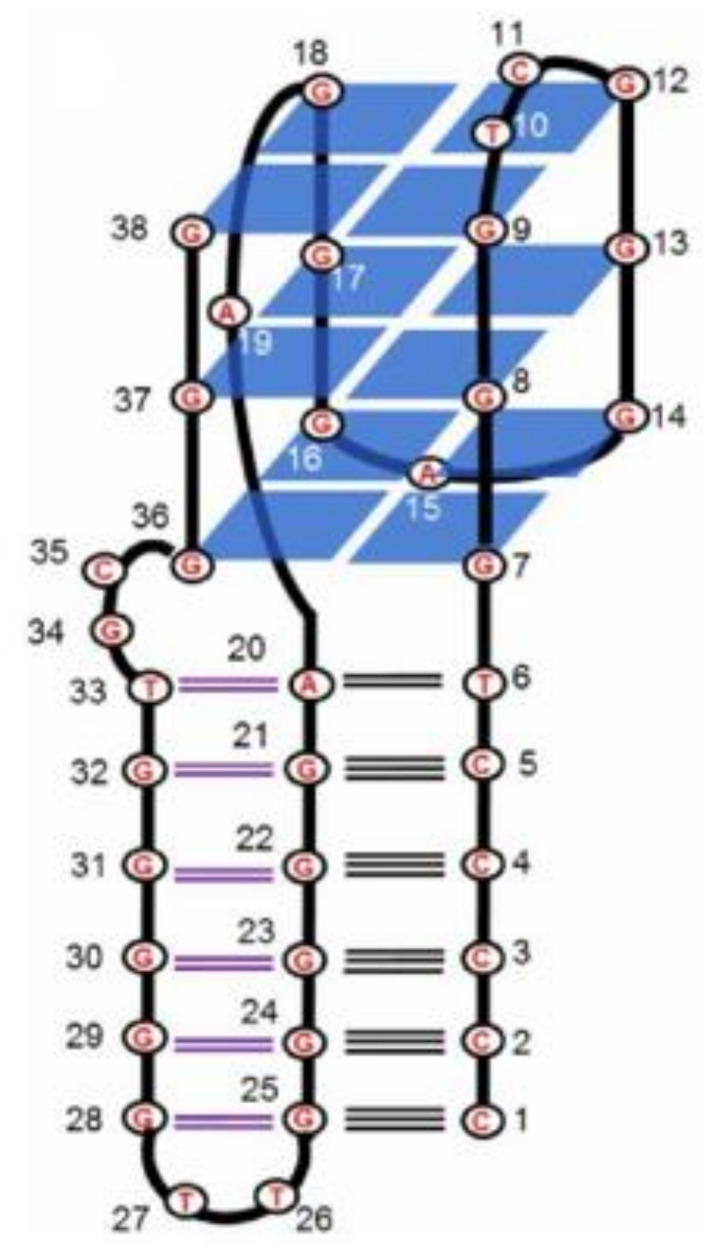
Schematic structure of CBA-1 [70]. Reproduced under the Creative Commons Attribution License.

An interesting example of quadruplex–triplex junction is the codeine-binding aptamer (Figure 19; Table 1, CBA-1) [70]. This oligonucleotide is composed of six guanosine tracts and one cytidine tract and folded into a G-quadruplex connected to a G·GC triplex. In the course of the conducted research, it was shown that codeine binding facilitates the formation of stable structures and increases the thermal stability of the quadruple–triplex junction. Moreover, it was also revealed that the compound preferentially adheres to the quadruplex–triplex junction, which could be assigned as the codeine-binding region or pocket. Based on the CD spectra analysis, it was proven that CBA-1 folded into a hybrid G-quadruplex (one antiparallel and three parallel strands) and triplex structure. Further analysis with the application of aptamer mutagenesis conferred that the presence of both structural elements is essential for codeine binding. Interestingly, based on knowledge of the natural occurrence of G-quadruplexes and triplexes in cells and their ability to terminate gene transcription, the authors developed an interesting experiment to mimic this process using DNA polymerase I, primer sequence, and splitting the CBA-1 into two strands. The 3′-termini of the aptamer was connected to the DNA template, whereas the remaining part of the CBA-1 was free in solution. The codeine-induced formation of the quadruplex–triplex junction was able to block polymerase reaction, what could have potential therapeutic applications. 

## 6. Conclusions

G-quadruplex-based aptamers constitute a large and promising group of nucleic acid-based molecular tools with high therapeutic potential. Interestingly, the biological activity of molecular tools based on G-quadruplexes can be successfully improved by adding additional structural elements. To date, the majority of structural modifications concerns quadruplex–duplex and quadruplex–quadruplex junctions (Table 1). Based on extensive literature data, it can be concluded that the vast majority of these junctions are of DNA type and mainly concern aptamers targeted towards thrombin. The duplex domain undoubtedly plays an important role in the folding of the G-quadruplex motif and in its activity. In general, the presence of a helix very often decreases the thermal stability of the G-quadruplex, however, simultaneously improves contact with the surface of the target protein. Furthermore, the length and sequence of duplex motif and linkers, which span both structural motifs, should be taken into account as being essential for activity when designing this type of junctions. 

Another very interesting group of junctions studied to date is the quadruplex–quadruplex connection. In this approach, two strategies can be recognized, i.e., the junction of two different G-quadruplex domains or the connection of two G-quadruplex motifs of the same type. In the case of the first option, both domains are usually targeted towards different active sites of the same molecule increasing the bivalency of the junction. This solution results in multiple binding events and improved target selectivity. On the contrary, the junction of two identical G-quadruplexes leads to novel aptamer targeted towards the same binding site. In such a situation, one domain is responsible for the inhibitory potential of the junction, whereas the second one controls the action of the first one. Furthermore, the composition and length of the linker, which joins both G-quadruplex motifs, is essential for the activity of the quadruplex–quadruplex junctions. Importantly, the superior biological activity of the junctions can be successfully achieved by the application of covalently bound linkers as well as of the non-covalent junction of two G-quadruplex domains.

A few articles describing other than a duplex or G-quadruplex connection with G-quadruplexes indicate that additional structural elements such as a triplex or hairpin clearly influence the thermal stability and biological activity of the G-quadruplex domain. Moreover, similar to other structural motifs, also here the composition and/or length of an additional domain plays an important role in the folding and inhibitory activity of the whole junction.

The promising physicochemical and biological characteristics of G-quadruplex junctions with other structural motifs studied so far provide preliminary evidence for the favorable modification of G-rich structures. Undoubtedly, the connection of G-quadruplexes with helices, hairpins, triplexes, or other G-quadruplexes appear favorable from the therapeutic point of view, thus increasing the repertoire of molecules potentially useful in the therapy of human diseases. The doors for the development of new molecular tools based on nucleic acids have been already opened. However, the area of research discussed herein still requires detailed studies on the requirements needed to design G-quadruplex junctions with superior properties. Importantly, the combination of structural and chemical modifications of G-quadruplex-based junctions and their properties are still mostly a blank slate, thus leaving a great scope of activity for future investigations.

## Figures and Tables

**Table 1:** Overview of the G-quadruplex junctions.

Name	Sequence (5′-3′) ^#^	Type of Junction	Target	Stability *	Biological Activity *	Reference
ONM	N_5_N_0-3_ **GGN_1-2_TGGN_2-5_N_0-3_GGTN_1-2_GG** N_0-3_N_5_	Q4/dpx	Thrombin	Higher	Higher	[52]
ONK	CACTGN_3_**GGTTGGTGTGGTTGG**N_3_CAGTG	Q4/dpx	Thrombin	Higher/lower	Higher/lower	[53]
31TBA	CACTGGTA**GGTTGGTGTGGTTGG**GGCCAGTG	Q4/dpx	Thrombin	Comparable to TBA	Higher	[31]
HD22-29 (60-18(29))	AGTCCGT**GGTAGGGCAGGTTGG**GGTGACT	Q4/dpx	Thrombin	n.d.	Lower	[54]
RE31	GTGACGTA**GGTTGGTGTGGTTGG**GGCGTCAC	Q4/dpx	Thrombin	Higher/similar	Higher	[30,55]
NU172	CGCCTA**GGTTGGGTAGGGTGG**TGGCG	Q4/dpx	Thrombin	Lower	Higher	[56,57]
AS1411-N6	GGTTGG **GGTGGTGGTGGTTGTGGTGGTGGTGG** CCAACC	Q4/dpx	Nucleolin	n.d.	Lower affinity	[34]
Aptamer I	AGTCCGT**GGGTAGGGCGGGTTGGG**GGTGACT	Q4/dpx	Hemin	n.d.	Higher affinity and activity	[58]
Aptamer II	AGTCCGT**GGTTGGTGTGGTTGG**GGTGACT	Q4/dpx	Thrombin/Hemin	n.d.	Higher affinity and activity	[58]
Aptamer III	**AGGGACGGGAAG**AAAAGGAAAATCCTTAAAAT**GTGGAGGGT**	Q4/hairpin	Hemin	n.d.	Higher affinity and activity	[58]
THR5	AGTCCGTGGTAGGGCAGGTTGGGGTGACT *(Spacer18)5* GGTTGGTGTGGTTGG	Q4/Q4	Thrombin	n.d.	Higher	[59]
THR6	AGTCCGT**GGTAGGGCAGGTTGG**GGTGACT *(Spacer18)10***GGTTGGTGTGGTTGG**	Q4/Q4	Thrombin	n.d.	Higher	[59]
THR7	**GGTTGGTGTGGTTGG***(Spacer18)10* AGTCCGT**GGTAGGGCAGGTTGG**GGTGACT	Q4/Q4	Thrombin	n.d.	Higher	[59]
HD1-15dA-60.29	**GGTTGGTGTGGTTGG***AAAAAAAAAAAAAAA*AGTCCGT**GGTAGGGCAGGTTGG**GGTGACT	Q4/Q4	Thrombin	n.d.	Higher	[60]
HD1-5dA-60.29	GGTTGGTGTGGTTGG*AAAAA*AGTCCGTGGTAGGGCAGGTTGGGGTGACT	Q4/Q4	Thrombin	n.d.	Higher	[60]
Linker 0	**GGTTGGTGTGGTTGG**AGTCCGT**GGTAGGGCAGGTTGG**GGTGACT	Q4/Q4	Thrombin	n.d.	Higher	[61]
Linker 5	**GGTTGGTGTGGTTGG***TTTTT*AGTCCGT**GGTAGGGCAGGTTGG**GGTGACT	Q4/Q4	Thrombin	n.d.	Higher	[61]
Linker 10	**GGTTGGTGTGGTTGG***TTTTTTTTTT*AGTCCGT**GGTAGGGCAGGTTGG**GGTGACT	Q4/Q4	Thrombin	n.d.	Higher	[61]
Linker 20	**GGTTGGTGTGGTTGG***TTTTTTTTTTTTTTTTTTTT*AGTCCGT**GGTAGGGCAGGTTGG**GGTGACT	Q4/Q4	Thrombin	n.d.	Higher	[61]
Bi-4S	GTCCGTG**GTAGGGCAGGTTGG**GGTGACT*(S)4T*GGTTGGTGTGGTTGG	Q4/Q4	Thrombin	n.d.	Lower	[34]
Bi-6S	GTCCGT**GGTAGGGCAGGTTGG**GGTGACT*(S)6T*GGTTGGTGTGGTTGG	Q4/Q4	Thrombin	n.d.	Higher	[34]
Bi-8S	GTCCGT**GGTAGGGCAGGTTGG**GGTGACT*(S)8T***GGTTGGTGTGGTTGG**	Q4/Q4	Thrombin	n.d.	Higher	[34]
Bi-10S	GTCCGT**GGTAGGGCAGGTTGG**GGTGACT*(S)10T***GGTTGGTGTGGTTGG**	Q4/Q4	Thrombin	n.d.	Higher	[34]
RNV220	**GGTTGGTGTGGTTGG***TEG*AGTCCGT**GGTAGGGCAGGTTGG**GGTGACT*inv-dT*	Q4/Q4	Thrombin	n.d.	Higher	[62]
RNV220T	**GGTTGGTGTGGTTGG***TTTT*AGTCCGT**GGTAGGGCAGGTTGG**GGTGACT*inv-dT*	Q4/Q4	Thrombin	n.d.	Higher	[62]
RA-36	**GGTTGGTGTGGTTGG** *T* **GGTTGGTGTGGTTGG**	Q4/Q4	Thrombin	Lower	Higher	[63,64]
AA	**GGTTGGTGTGGTTGG** *AA* **GGTTGGTGTGGTTGG**	Q4/Q4	Thrombin	Higher	Higher	[64]
TT	**GGTTGGTGTGGTTGG** *TT* **GGTTGGTGTGGTTGG**	Q4/Q4	Thrombin	Higher	Higher	[64]
Gly	**GGTTGGTGTGGTTGG** *Gly* **GGTTGGTGTGGTTGG**	Q4/Q4	Thrombin	Higher	Lower	[64]
AGlyA	**GGTTGGTGTGGTTGG** *AGlyA* **GGTTGGTGTGGTTGG**	Q4/Q4	Thrombin	Higher	Higher	[64]
TGlyT	**GGTTGGTGTGGTTGG** *TGlyT* **GGTTGGTGTGGTTGG**	Q4/Q4	Thrombin	Lower	Lower	[64]
GS-522	**GGTTGGTGAGGTTGG**	Q4/Q4	Thrombin	n.d.	n.d.	[65]
cTU1	**pGAGTCCGTGGTAGGGCAGGTTGGGGTGACTCGCTGTGGTTGGTGAGGTTGGCAGC** (ligated)	Q4/Q4	Thrombin	Higher	Higher	[65]
cTU2	**pGAGTCCGTGGTAGGGCAGGTTGGGGTGACTCGCTGTGGTTGGTGAGGTTGGACAG** (ligated)	Q4/Q4	Thrombin	Higher	Higher	[65]
cTT-2	**pGCTGTGGTTGGTGAGGTTGGCAGCGCACTGGTTGGTGAGGTTGGGTGC** (ligated)	Q4/Q4	Thrombin	Higher	Higher	[65]
cTTTT-1	**pAATTCGTAGCTCCAGTGTGTGGTTGGTGAGGTTGGCACACTGGACGATGCTGTGGTTGGTGAGGTTGGCAGCATCGCCGCTACGpAATTCGTAGCTCCAGTGTGTGGTTGGTGAGGTTGGCACACTGGACGATGCTGTGGTTGGTGAGGTTGGCAGCATCGCCGCTACG **(ligated)	Q4/Q4	Thrombin	Higher	Higher	[65]
HD1-ds	(**GGTTGGTGTGGTTGG**TTCGAACGTGACATCG)/(**GGTTGGTGTGGTTGG**TTCGATGTCACGTTCG)	Q4/Q4	Thrombin	Lower	Slightly lower	[35]
T4T4	(**GGTTGGTGTGGTTGGTTTTGGGGTTTTGGGG**)_2_	Q4/Q4	Thrombin	Lower	Slightly lower	[35]
FF	**(GGTTGGTGTGGTTGG** *TTTTT* TTTTTTTTTTTTTTT **)** **(** AAAAAAAAAAAAAAA *TTTTT* **GGTTGGTGTGGTTGG)**	Q4/Q4	Thrombin	Lower	Higher	[66]
FH	**(GGTAGGGCAGGTTGG***TTTTT*TTTTTTTTTTTTTTT**)** (AAAAAAAAAAAAAAA*TTTTT***GGTTGGTGTGGTTGG)**	Q4/Q4	Thrombin	Lower	Higher	[66]
R24	**(GGAGGAGGAGGA)**_2_	Q4/Q4	Prion protein	n.d.	Higher	[67]
R12-A-R12	**(GGAGGAGGAGGA)** *A* **(GGAGGAGGAGGA)**	Q4/Q4	Prion protein	n.d.	higher	[67]
RT5	ATC(CGCCTGATTAGCGATACTCAGGCG)CC**GGGGGGGTGGG**AATACAGTGATCAGCGACTTGA GCAAAATCACCTGCA**GGGG**	Q4/hairpin	HIV-1 reverse transcriptase (RT)	n.d.	n.d.	[68]
RT6	ATC(CGCCTGATTAGCGATACTCAGGCG)TTA**GGGAAGGG**CGTCGAAAGCA**GGGTGGG**ACTTGA GCAAAATCACCTGCA**GGGG**	Q4/hairpin	HIV-1 reverse transcriptase (RT)	n.d.	n.d.	[68]
RT47	ATC(CGCCTGATTAGCGATACTCAGGCC)TT**GGGCGGGCCGGG**ACAATGGAGAGATTTACTTGA GCAAAATCACCTGCA**GGGG**	Q4/hairpin	HIV-1 reverse transcriptase (RT)	n.d.	n.d.	[68]
Myc-dup3	TGA**GGGTGGGTAGGGTGGG**(CTAGTCATTTTGACTAG)	Q4/hairpin	Indoloquinoline ligand	n.d.	n.d.	[69]
Myc-dup5	(GATCAGTTTTACTGATC)**GGGTGGGTAGGGTGGGTA**	Q4/hairpin	Indoloquinoline ligand	n.d.	n.d.	[69]
CBA-1	CCCCCT**GGGTCGGGAGGGA**AGGGGGTTGGGGGTGC**GGG**	Q4/triplex	Codeine	n.d.	n.d.	[70]

* in comparison to regular, naked G-quadruplex; **^#^** G-tracts are shown in bold and complementary tracts are underlined.

## Data Availability

Not applicable.
